# Multiscale visualization of the structural and characteristic changes of sewage sludge biochar oriented towards potential agronomic and environmental implication

**DOI:** 10.1038/srep09406

**Published:** 2015-03-24

**Authors:** Jining Zhang, Fan Lü, Hua Zhang, Liming Shao, Dezhen Chen, Pinjing He

**Affiliations:** 1 State Key Laboratory of Pollution Control and Resource Reuse, Tongji University, Shanghai 200092, China; 2 Institute of Waste Treatment and Reclamation, Tongji University, Shanghai 200092, China; 3 Centre for the Technology Research and Training on Household Waste in Small Towns & Rural Area, Ministry of Housing and Urban-Rural Development of P.R.China (MOHURD), Shanghai 200092, China; 4 Thermal and Environmental Engineering Institute, Tongji University, Shanghai 200092, China

## Abstract

Sewage sludge biochars were obtained at different pyrolysis temperatures from 300°C to 900°C and their macro- and microscale properties were analyzed. The biochar's plant-available nutrients and humus-like substances in the water-extractable phase and fixed nutrients in the solid fraction were evaluated for their potential agronomic implications. FT-IR, Raman, XRD, XPS, and SEM techniques were used to investigate the chemical structure, functional groups, and microcrystal structure on the surface of the biochar. The results revealed minor chemical changes and dramatic mass loss in the biochar obtained at 300–500°C, whereas significant chemical changes in the biochar were obtained at 600–900°C. The concentrations of plant-available nutrients as well as fulvic- and humic-acid-like materials decreased in the biochar samples obtained at higher temperatures. These results implied that the biochar samples pyrolyzed at 300–500°C could be a direct nutrient source and used to neutralize alkaline soil. The surface area and porosity of the biochar samples increased with temperature, which increased their adsorption capacity. Rearrangement occurred at higher temperature 600–900°C, resulting in the biochar becoming increasingly polyaromatic and its graphite-like carbon becoming organized.

Biotreatment of municipal and industrial wastewaters unavoidably leads to significant quantities of sludge. For sewage sludge treatment, pyrolysis is a promising pathway^1^,^2^,^3^. Pyrolysis of sewage sludge is the thermal degradation of sludge in the absence of air or in an oxygen-deficient atmosphere, transforming the sludge organic matter into biogas, bio-oil, and carbonaceous biochar residue. When compared with raw sludge, sludge biochar has minimum pathogens and odor, and is capable of concentrating heavy metals^4^ (except mercury and cadmium). Studies comparing sludge biochar with raw sludge revealed that the soil properties were improved after applying sludge biochar to the soil as amendment^5^,^6^,^7^. Therefore, there has been increasing interest in the use of sludge biochar for soil beneficiation and carbon sequestration in a long-lasting solid form. The agronomic performance and effectiveness of biochar, such as available nutrients, is essentially depend on its characteristics, and the physical and chemical properties of sludge biochars are strongly influenced by the pyrolysis conditions, predominantly by the highest treatment temperature (HTT).

During the pyrolysis process, sludge biochar undergoes various physical, chemical, and molecular changes. Some of the documented changes include those in the yield, volatile contents, pH, electrical conductivity (EC), hardness, bulk density, and element composition that are related to macro-nutrient benefits for agro-application^1^,^8^,^9^. Whereas, the other part of studies have focused on the behavior of heavy metals during low-temperature pyrolysis of sewage sludge at 500°C^4^, 300–700°C^10^, 300–500°C^11^, and 400–450°C^12^, which are related to the toxicity for agro-application. These two parts of researches were rarely put together and balanced the pros and cons of sludge biochar simultaneously. Furthermore, concentrations of heavy metals might decrease at higher temperature^13^. Therefore, it arouses the research demand on higher temperature scenario and the systematic comparison on a wider temperature range (e.g. 300–900°C in the present study). Meanwhile, the existing studies on from which pyrolysis temperature the biochar could be much beneficial is ambiguous and inclusive. For example, reported^14^ that biochar obtained at 600°C was more effective in reducing CO_2_ emission from soil, when compared with that obtained at 400°C. Whereas, Song^12^ demonstrated that garlic planted in soil amended with biochar obtained at 450°C contained the lowest level of heavy metals, when compared with that planted in soil amended with other biochars obtained at 500–550°C. This uncertainty of biochar application should be attributed to the insufficient description about their studied biochar, i.e. only macro-scale characteristics were recorded. In fact, the external properties are essentially determined by the intrinsic structure and composition of biochar samples. Hence, it is necessary to investigate the macro- and microscale properties of the final sewage sludge biochar, and then differentiate them systematically for easier categorization and for better evaluating the potential agronomic application of sludge biochar based on the above investigation.

Therefore, the present study was to gain more systematic insight into how pyrolysis temperature in a wider range affects the multiscale characteristics of biochar samples, and to clarify the intrinsic relationships between sludge-derived biochar and the development of surface microstructure during the heating process. Not only investigating the above-mentioned bulk parameters, but also visualizing the surface microstructure of sludge biochar, as well as discussing the biochar's resultant pros and cons potentials for applications was the aims of this study.

## Results

### Physicochemical analysis

The physicochemical characteristics and elemental composition of the biochar samples produced at different pyrolysis temperatures are listed in Table 1. The biochar yield and heating value decreased with the increasing pyrolysis temperature. The yield of biochar dropped from 64.28 to 46.66 wt% of the dry mass when the pyrolysis temperature was increased from 300°C to 700°C. However, only an additional 4.44 wt% yield was reduced when the temperature was increased from 700°C to 900°C. The decrease in the biochar yield with the increasing pyrolysis temperature could possibly be related to the cracking and volatilization process, which was also reported^8^ for sludge biochar pyrolyzed at different temperatures of 450°C, 650°C, and 850°C. The heating value of the biochar dropped linearly from 11054.3 to 6635.6 kJ kg^−1^ when the pyrolysis temperature was increased from 300°C to 600°C, and then almost remained constant with the temperature increasing from 600°C to 900°C.

An increase in the pyrolysis temperature from 300°C to 900°C led to an increase in the fixed carbon (FC) content from 15.75 to 21.25 wt%, and an increase in the ratio of FC to carbon from 42.4 to 84.2. The FC content represented the efficiency of the pyrolytic conversion of ash-free organic matter in the sludge to a relatively pure, ash-free carbon^15^. The highest ash content and lowest volatile matter content were found in biochar sample of 900°C, which was mostly due to volatilization accompanied by the accumulation of inorganic oxides such as Si, Al, and Fe^16^. For instance, the XRF results revealed that the ash composition of the biochar samples comprised 19.1%–22.7% Si. Comparison of the Si, Al, Ca, and Fe contents of the ash composition (Table 2) indicated the increase in the inorganic components with the increasing pyrolysis temperature. One of the main characteristics of sewage sludge is the presence of high amounts of inorganic ash, when compared with other materials such as wood biochar or lignocellulosic char obtained from agricultural waste^1^. The increase in the amount of inorganic ash in the biochar could increase its mineral composition and capacity to adsorb polar molecules.

### Carbon, hydrogen, oxygen, nitrogen, and sulfur

The variations in the results of the elemental analyses of the sludge biochar samples with temperature are shown in Table 1. The biochar samples showed the pattern of depleted content of some elements. In samples of 300–900°C, the carbon content decreased from 37.15 to 25.23 wt% of dry mass, the hydrogen content decreased from 4.35 to 0.64 wt%, the nitrogen content decreased from 6.17 to 1.24 wt%, the sulfur content decreased from 1.54 to 0.55 wt%, and the oxygen content decreased from 13.37 to 1.16 wt%. Particularly, when compared with the original dried sludge, more than 67.5% of hydrogen was removed in sample at 500°C, and more than 80.0% of hydrogen was lost in samples pyrolyzed at over 700°C. Moreover, the H/C and O/C atomic ratios of the biochar samples demonstrated a decreasing trend with increasing pyrolysis temperature. Lower pyrolysis temperatures (samples at 300–500°C) resulted in a higher H/C ratio (1.41–0.67) and O/C ratio (0.27–0.15), whereas higher temperatures (samples at 600–900°C) presented lower H/C ratios (0.51–0.30) and O/C ratios (0.15–0.03). When compared with the biochar samples pyrolyzed at lower temperatures, those obtained at higher temperatures were less polar and had greater aromaticity and carbonization.

### Nutrients and trace metals of biochar samples in solid form

Table 3 showed the total contents of trace metals in raw sludge and sludge biochar samples measured by wet acid-extract method followed by ICP in accordance with EPA method 3050B. The contents of majority of trace metals, such as Al, Fe, and Zn in the final residue were greater than those in their feedstock sludge, which showed that pyrolysis process condensed and retained these trace metals in the final residue. Furthermore, the enrichment effect became more evidently with pyrolysis temperature rising.

For comparison purposes, we also determined the total amounts of heavy metals in solid form using XRF technique. The results showed that all heavy metals have their total concentration increasing with temperature (Supplemental information: Table S1). Thus, we can find a bit difference between two dataset. It should be noticed firstly that, to gain insight into the precision of the heavy metal analyses between XRF technique (sample non-destructive method) and acid-extraction method (sample destructive method), comparison is difficult when different extraction techniques are used^17^. Only much could be learned that most of the heavy metals are retained in the sludge biochar samples.

Secondly, incomplete recovery might happen for acid-extraction method. As pointed^18^, acid digestion and thermal decomposition steps can result in analyte losses, incomplete recoveries, and/or sample contamination; USEPA Method 3050 is unsatisfactory for some elements; Variations in hot plate temperatures, refluxing times and acid additions directly affect elemental recoveries; the observed relative standard deviations of 10–30% for this method are considerably greater than the expected instrumental error (<5%) for an ICP. Therefore, when samples of very trace amount were measured, the deviation will be significant. Thirdly, the total concentration of Cu and Mn increased at sludge biochar samples at 300–700°C, on the contrast, decreased at biochar samples at 800–900°C. Some researchers investigated the fate of heavy metal contents in the sewage sludge biochar samples with pyrolysis temperature, and found that the total concentration of Cu and Mn increasing with pyrolysis temperature; However, the selected pyrolysis temperature was from 300–700°C^10^; 300–500°C^11^; 250–700°C^19^ and so on. Little references were discussed on the variations of heavy metals in the sludge biochar samples over 700°C. Therefore, the reasons of Cu and Mn concentration determined by acid-extraction method decreasing at 800–900°C in this study were explained, 1) The Cu volatilizes to a significant extend around 800°C in case of sewage sludge incineration^20^; 2) Guo^21^,^22^,^23^ observed the Mn bleeding ratio could be above 10% during coal pyrolysis. Specially, they found that the bleeding ratio of all the studied elements increased sharply when the coal pyrolysis temperature increased from 700°C to 800°C; 3) The existence of chloride could accelerate the volatile of Cu and Mn. Although the Cl concentration was not analyzed in this study, sewage sludge is generally regarded to contain Cl compounds owing to some Cl-containing conditioners during sludge dewatering or to some Cl-containing surfactant in sewage. Actually, reported^17^ that the Cl concentration was 3.6 mg g^−1^ and 4.0 mg g^−1^ in the studied raw sewage sludge and sludge biochar samples, and it reached 5.4 mg g^−1^ in the studied sludge compost^24^. So, as a result of the existence of Cl in the biochar samples, it could be incorporated with Cu and Mn, and their chlorides would be easy to be released to the gas in the form of CuCl_2_ and MnCl_2_. The melting points of CuCl_2_ and MnCl_2_ are 498°C and 650°C in the pure samples; they would be over 700°C in the multi-composition and complicated sludge biochar system in this study.

### Water-extractable fractions of the biochar samples

The agronomic availability of biochars primarily depends on the initial water-extractable nutrient contents (Table 4). Although dissolved organic matter (DOM) represents a small proportion of organic matter residue in the biochar, it is significant in the soil amendment/ecosystem owing to its mobility and reactivity^25^. The DOC and DN indicate the DOM contents. In the present study, the DOC contents decreased rapidly from 24.23 mg g^−1^ in sample at 300°C to 2.66 mg g^−1^ in sample at 400°C, and then reduced to almost zero in samples of 600–900°C. This was due to secondary reactions, which resulted in low molecular weight acids and neutral compounds, which were dominant in the biochars at higher temperatures^25^. The DN and NH_4_^+^-N have important agronomic uses because they are the main sources of nitrogen available for plant uptake. Their contents in sample at 300°C were 6.19 and 4.39 mg g^−1^, respectively, which decreased to 0.57 and 2.15 mg g^−1^, respectively, in sample at 400°C, and then reached almost zero or below the detection limit in samples of 500–900°C. Reported^1^ that the available nitrogen content in the form of DN and NH_4_^+^-N was higher in the sludge biochar samples produced at lower pyrolysis temperatures (<400°C).

The contents of water-extractable K, Na, P, and Mg reduced rapidly with the increasing pyrolysis temperature, and when compared with the raw sludge, almost 90% of the water-extractable K, Na, and P contents and 30% of Mg content were lost in sample at 400°C. Similarly, the water-extractable Ca content in the biochar samples also presented a downward trend, when compared with the raw sludge.

**Figure 1:**
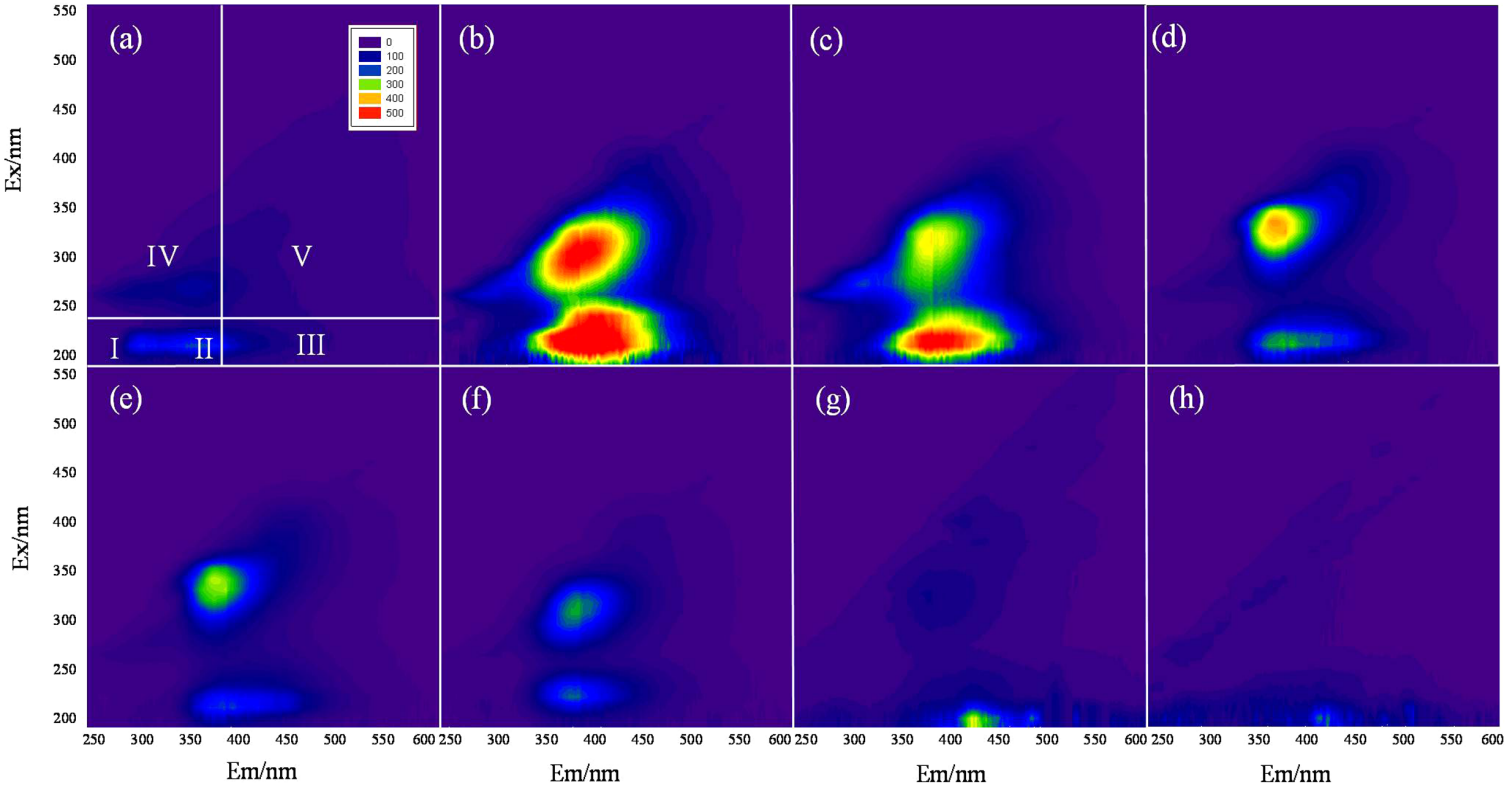
EEM spectra of the sewage sludge and sludge biochar samples at various temperatures. (a) primary sludge; (b) C300; (c) C400; (d) C500; (e) C600; (f) C700; (g) C800; (h) C900.

Three-dimensional fluorescence EEM was used to study the aqueous humus-like compounds generated in the sludge biochar samples. Although EEM is widely applied in compost and soil research to detect protein- or humus-like organic matters, a limited number of studies had used this technique to analyze biochar with respect to temperature. The EEM spectra, normalized to the DOC content for the primary sludge and sludge biochar samples, are presented in Fig. 1. An EEM spectrum could be divided into four excitation-emission regions: Region I (Ex < 250 nm; Em < 380 nm), protein-like organic compounds; Region II (Ex < 250 nm; Em > 380 nm), fulvic-acid-like materials; Region III (Ex > 250 nm; Em < 380 nm), soluble microbial byproduct-like materials; and Region IV (Ex > 250 nm; Em > 380 nm), humic-acid-like materials.

In the contour of raw sludge itself, organic compounds were found to be composed of aromatic proteins (Region I) and soluble microbial byproduct-like materials (Region III). The volumes of fulvic-acid-like materials (Region II) and humic-acid-like materials (Region IV) were low. The scope and intensity of the fluorescence area were the highest for the biochar sample at 300°C, followed by samples at 300–500°C, and eventually became undetectable at higher temperatures (700–900°C). These results suggested that the sludge biochar produced at lower temperatures (300–500°C) had more fulvic- and humic-acid-like materials.

The pH and EC values of the biochar samples are listed in Table 1. The pH values of the biochar samples at 300–800°C ranged from 6.2 to 11.9, and then decreased to 9.4 for sample at 900°C. The EC values correspond to the concentration of total dissolved salts and could be used to describe the variation in the organic and inorganic ions. The EC value of the primary sludge was 4.7 ds m^−1^, which decreased to 0.3–0.4 ds m^−1^ with the increasing pyrolysis temperature. However, regardless of the trend of the pH and EC results, sample at 700°C presented higher values. Higher pyrolysis temperatures led to higher pH of the biochar samples, which was mainly dueto the minerals present in the biochar samples and the increase in their contents during the pyrolysis process. Thus, the accumulation of these basic cations increased the pH values of the biochar samples^26^,^27^,^28^. Furthermore, the concentration of Ca^2+^ increased with the increasing pH of the biochar, while the total content of K^+^ and Na^+^ increased with the increasing EC value of the biochar, which are in agreement with the previous results^28^.

The accumulation of trace heavy metals are of great concern in agricultural product due to potential threat for human and animal health. As listed in Table 3, most of trace metals in the biochar samples were greater at higher pyrolysis temperatures than those at lower temperatures. To know the bioavailability of these metals contained in solid form, we also investigated the total concentrations for trace metals in water-extractable solutions. Fortunately, no detection contents for water extractable trace metals were observed in the studied sludge biochar samples (Table 4). It meant that the metals in sludge biochars were in fixed form. The metal suppression did not only depend on the neutral to alkaline buffer properties of the biochar, but also depend on the biochar pore structure and BET surface, which enhance biochar ability to immobilize heavy metal^19^. Those implied that the biochar generated at 300–900°C may have minimal impact on increasing the compost/soil heavy metal contents following a single short-term application.

### XRD spectra

The XRD spectra of the biochar samples are shown in Fig. S2 (Supplemental information: Fig. S2). The analysis of the XRD patterns revealed the presence of several mineral phases. Quartz, with a characteristic peak at 2θ = 26.6°, was the most recognizable crystallographic structure at all temperatures. The sharpness of the peak increased with the increasing temperature, possibly owing to the ultrastructural changes in the sludge biochar. Calcite (CaCO_3_) and dolomite [CaMg(CO_3_)_2_] were detected in the biochar samples at 300–800°C, while carbonates underwent decomposition and were not present at higher temperatures (>800°C). Some amount of Ca, which was present as CaCO_3_ in samples heated at 700°C, decomposed to CaO during high-temperature pyrolysis^1^,^27^. This was also a reason for the higher pH values of the samples pyrolyzed at 700–800°C, and the samples' basicity was mainly linked to the presence of Ca.

### BET surface area and SEM morphology

The BET surface areas of the raw sludge and biochar samples are listed in Table 1. The BET surface area of the as-received sludge was considerably low (2.88 m^2^ g^−1^). However, the surface area of the sludge biochar linearly increased with the increasing pyrolysis temperature from 4.88 m^2^ g^−1^ (at 300°C) to 19.11 m^2^ g^−1^ (at 800°C). At 900°C, the BET surface area of the biochar increased substantially up to 34.12 m^2^ g^−1^.

The SEM general morphology (Supplemental information: Fig. S3) of the biochar samples also exhibited an increased surface area with the increasing pyrolysis temperatures. The SEM images of the as-received sludge indicated plate-like layer construction and poor structure that was smoothly compacted (Fig. S3a). However, as shown in Fig. S3b, a crack appeared and tar agglomerates seemed to cover the surface of the biochar particle. Furthermore, in the biochar samples, the dense and tightly packed microstructure disintegrated, gradually forming fragments (Fig. S3c–e), and a characteristic hollow was observed (Fig. S3f–h). On the one hand, lower temperature entailed condensation of organic volatiles, which could lead to pore clogging and reduction in the total surface area. On the other hand, at higher temperature, volatilization was more subtle, making the biochar more porous and creating voids within the biochar matrix.

### FT-IR and XPS spectra

Fig. S4 (Supplemental information: Fig. S4) presented the FT-IR spectra of the dried raw sludge and biochar samples. The absorption bands and peaks provided evidence of the presence of some surface functional groups. In general, the organic functional groups found in the biochar spectra decreased or even disappeared as a result of pyrolysis.

The broad band at 3400 cm^−1^ was assigned to hydroxyl (-OH) stretching, and the peak intensity decreased rapidly at samples 300–500°C, suggesting an ignition loss of -OH^10^. The peaks at 2925 and 2855 cm^−1^ corresponded to the aliphatic CH_3_ asymmetric and symmetric stretching vibration, respectively, which had been assigned to the fats and lipids of the sewage sludge^29^. These peak intensities decreased owing to the continuous decrease in the labile aliphatic compounds as well as demethylation and dehydration. The loss of -OH and aliphatic groups as well as a concurrent development of fused-ring structures gave rise to pore formation. These results were consistent with the SEM findings. The peaks at 1650 cm^−1^ were assigned to the amide I bands of protein origin. These bands gradually broadened and shifted towards lower wavenumbers as a result of pyrolysis. The decomposition of protein mainly occurred at 300–400°C^30^, which could be explained by a decrease in the amide groups and simultaneous increase in the amino acid functionalities. The band at 1430 cm^−1^ became invisible, and this wavenumber had been assigned to the stretching of C in the heteroaromatic structures^31^. The sharp peak at 1030 cm^−1^ was assigned to C-O stretching of polysaccharides or polysaccharide-like substances. This peak decreased at higher pyrolysis temperature and appeared as a shoulder for the biochar samples at 400–800°C, and eventually became invisible for the sample at 900°C. Meanwhile, a peak at 1080 cm^−1^, which was present in a similar position in the broad region, was assigned to Si-O, indicating the presence of silicate impurities and clay minerals. Si was noted to be one of the major inorganic constituents in the sludge biochar samples, which was verified by the XRF results (Table 2).

Overall, minor chemical changes occurred at lower temperature, and most of the spectral features were lost and the spectrum began to resemble pure graphite over 700°C. The FT-IR results indicated that the rearrangement continued to occur at higher temperatures, resulting in the sludge biochar becoming increasingly polyaromatic in nature.

The nitrogen gradually transformed into pyridine-like structure occurring in heterocyclic compounds with the increasing temperature, which was confirmed by the peak extraction of N regions in the XPS spectra. Three binding energies of 398.7, 400.4, and 401.1 eV corresponded to pyridine nitrogen (N-6), pyrrolic nitrogen (N-5), and quaternary nitrogen (N-Q), respectively. The integrated areas of the individual components were calculated, and are shown in Table 5. The fractions of N-5 and N-Q were the main components of the raw sludge, of which the integrated area of N-5 accounted for 61.1%. As the pyrolysis temperature increased, the fraction of N-6 increased. The conversion of N-5 to N-6 and N-Q under pyrolysis had already been demonstrated by Schmiers^32^. N-6 and N-Q were the most stable forms of nitrogen binding at higher temperatures. The pyridinic ring was preferentially incorporated into the graphitic-like carbon structure in the form of quaternary nitrogen^32^, which affected the characteristics of the sludge biochar, including biochar basicity and available nitrogen forms^33^,^34^.

### Raman spectra

Raman spectrum was also used to further analyze the structure of the carbon materials, and the results of the Raman spectra in the region from 0 to 2000 cm^−1^ are shown in Fig. S5 (Supplemental information: Fig. S5). All of the curves exhibited two relatively broad Raman bands at Raman shifts at 1350 and 1580 cm^−1^, which corresponded to the D-band and G-band, respectively. The D-band could be labeled as the amorphous or disordered graphite, while the G-band could be regarded to indicate the presence of graphitic crystallites. The graphitic degree of carbons was confirmed by the value of I_D_/I_G_^35^,^36^. The I_D_/I_G_ ratios calculated by the Gaussian function of each sample were 8.3, 7.5, 5.7, 4.9, 4.2, 3.8, and 6.4, and the values were found to decrease gradually with increasing pyrolysis temperature. Kwiecinska^37^ demonstrated that the I_D_/I_G_ results (3.1–0.0) decreased from greenschist facies to granulite facies graphite, indicating the increase in crystallographic order. Rhim^35^ reported an increase in the I_D_/I_G_ ratio (0.0–2.6) with the increasing pyrolysis temperature from 300°C to 650°C, and the subsequent decrease in the ratio (2.6–0.0) with the increasing temperature from 650°C to 2000°C by using microcrystalline cellulose as the carbonaceous sample. McDonald-Wharry^36^ obtained the I_D_/I_G_ ratio by using graphites and regular fullerenes, and found that an increase in the I_D_/I_G_ ratio (0.55–1.20) indicated a conversion of amorphous carbon to graphene-like domains. These variations in the I_D_/I_G_ ratios were mainly owing to the properties and carbon purity of the selected carbonaceous material. In fact, in the present study, trace oxygen concentration still remained even at high temperatures. These residual oxygen atoms were involved in the cross-linking of the carbon microstructure, yielding a non-graphitizing hard carbon^35^. However, the Raman spectra displayed characteristics that can be attributed to the carbonaceous materials with the least amount of structure order. In general, the growth and organization of aromatic clusters as well as carbon microstructure in the biochar samples became ordered and condensed at higher pyrolysis temperature.

## Discussion

**Figure 2:**
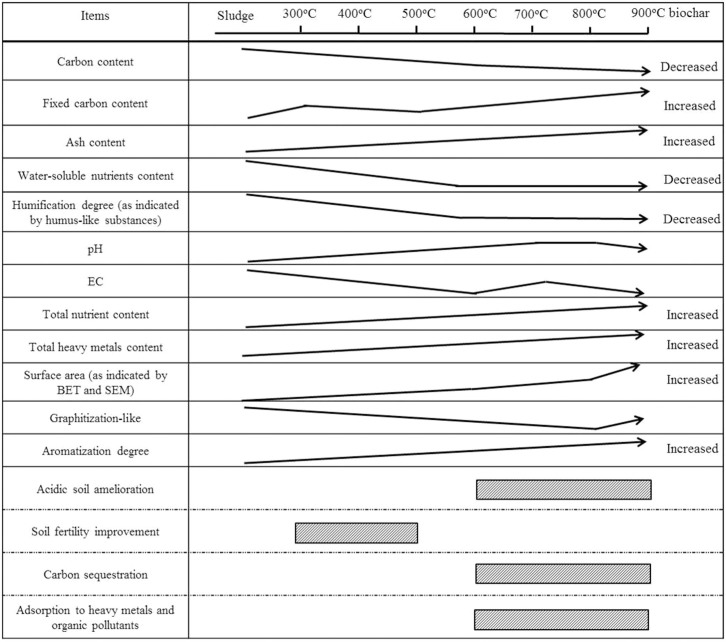
The classified properties and purposes of sludge biochar samples obtained at various temperatures.

Analysis of the acid-extractable and water-extractable fractions of the sludge biochar was conducted to evaluate the sludge biochar characteristics as well as visualize the microstructure of the solid form to describe the development on the surface of the sludge biochar. The pyrolysis process induced a significant change in the sludge-derived biochar carbon microstructure, and influenced the changes in the apparent characteristics of the biochar samples. As the sludge biochar samples exhibited widely varying properties, the present findings could help to select suitable biochar for different applications. The observed diversity of the biochar properties necessitates careful definition of the purpose of the agricultural applications of the biochar, such as pH amelioration, nutrient retention, or sequestration of soil organic matter, before selecting appropriate biochar. The multiple properties were classified and the purposes were summarized at Fig. 2.  (1) Some molecular arrangements and functional groups presented during the pyrolysis process were mainly composed of carboxyl groups, lactones, and phenols, all of which enhanced the capacity of the biochar to chemisorb nutrients, minerals, and DOM, in association with the surface oxidation capacity of the biochar^38^,^39^,^40^. The biochars obtained at higher temperatures, which developed a significant surface area, may improve the retention of nonpolar pollutants in soils or decrease the bioavailability of heavy metals to alleviate plant damage. The soils benefit more from biochars with high surface area, which are more efficient in increasing the water-holding capacity^41^ and reducing denitrification. Furthermore, owing to their high aromatization and recalcitrance, biochars could be stored in the field environment and could be helpful in effectuating C sequestration in soil.  (2) The concentration of water-extractable nitrogen, phosphorus, and potassium, particularly in its available form for plant uptake, was found to decrease with the temperature. Considering the biochars' yield, the results regarding the available form of nutrient elements and trace metals, as well as the presence of fulvic- and humic-acid-like compounds, lower temperature (300–500°C) could be deduced as suitable for biochar production. The mineral contents of biochar could play an important role in agronomic response even on a fertile soil. Hence, attention is needed when biochars with very low nutrient content is applied to agricultural soils, and supplementation with fertilizers may be required for adequate plant nutrition.  (3) The results obtained in the present study showed that if the soil intended for biochar application is acidic in nature, then the biochars produced at higher temperatures (600–900°C) could be used to neutralize the soil, because of their higher pH. Alternatively, biochars produced at lower temperatures might be suitable for alkaline soils to correct alkalinity problems.

## Methods

### Preparation of sludge biochar

Dewatered sewage sludge was collected from a local municipal wastewater treatment plant in Shanghai, China. The plant treats 75,000 m^3^ d^−1^ of wastewater (93% domestic and 7% industrial sewage) by using an anoxia–anaerobic–aerobic process. The sludge was dewatered by centrifugation with the addition of 3–5 wt% polyacrylamide as the flocculating agent. The dewatered sludge was incubated at 105°C in an oven to remove the residual water prior to pyrolysis. The primary properties of the as-received dried sludge are listed in Table 1.

All the pyrolysis processes were carried out in an electric furnace (YFFK10QK-GC, Shanghai, China) under nitrogen atmosphere and with a heating rate of 30°C min^−1^. The pyrolysis temperatures chosen were 300°C, 400°C, 500°C, 600°C, 700°C, 800°C, and 900°C, respectively, and these treatments are referred to C300, C400, C500, C600, C700, C800, and C900, respectively. All the as-received sludge biochar samples were crushed and sieved to a size of 2 mm for microscopic analysis and water extraction tests, as well as to a size of 75 μm for solid spectroscopic and elementary analyses.

### Proximate analysis

The yield of biochar was determined as the ratio of the weight of the produced biochar to the dry weight of the sewage sludge subjected to pyrolysis. The heating values were estimated using an oxygenbomb calorimeter (XRY-1A, Shanghai, China). The ash content was determined by dry combustion in a muffle furnace at 550 ± 10°C, and the volatile matter content was determined at 900 ± 10°C under air-free conditions. The fixed carbon (FC) content was estimated as follows: FC (%) = 100%−ash (%) −volatile matter (%).

### The pH, EC, concentrations of available nutrients and heavy metals in the water-extracted fraction

Water leaching tests were performed by shaking the samples with deionized water (1:10, w/v) at 200 rpm for 4 h in a horizontal shaker kept at room temperature. Thereafter, the pH of the supernatant was determined by using a pH electrode (pHS-2F, Shanghai, China), and the EC was ascertained by using an EC meter (DDS-307A, Shanghai, China).

The filtrate of each biochar sample was passed through a 0.45 μm polytetrafluoroethylene filter and the aqueous extracted fraction was analyzed. A total organic carbon analyzer (TOC-V_CPH_, Shimadzu, Japan) was used to measure the dissolved organic carbon (DOC) and dissolved nitrogen (DN). The ammonium nitrogen (NH_4_^+^-N) concentration was determined using a Kjeltec 8400 analyzer (Foss, Sweden). The dissolved phosphorous content was determined according to molybdate-antimony-scandium spectrophotometry method at the wavelength of 650 nm (UV-1800 spectrophotometer, Shimadzu, Japan). The concentrations of K^+^ and Na^+^ were measured by employing an atomic absorption spectrometer (AAnalyst 400, Perkin Elmer, USA). The Ca^2+^, Mg^2+^, and heavy metal contents were analyzed using inductive coupled plasma atomic emission spectroscopy analysis (Agilent 720ES, USA). Fluorescence excitation-emission matrix (EEM) spectroscopy analysis was subsequently applied by using a fluorescence spectrophotometer (Cary Eclipse, Varian, USA) in scan mode. A detailed account of the EEM analysis has been presented elsewhere^42^,^43^.

### Total concentration of nutrient elements and heavy metals

The dried and ground samples were subjected to acid digestion according to USEPA3050B using HF, HClO_4_, HNO_3_ and peroxide. The concentration of total phosphorus (TP) was determined using the molybdate-antimony-scandium spectrophotometry method at the wavelength of 650 nm (UV-1800 spectrophotometer, Shimadzu, Japan). The total concentration of K^+^ and Na^+^ were measured by employing an atomic absorption spectrometer (AAnalyst 400, Perkin Elmer, USA), while the other nutrient elements and metal contents were analyzed using inductive coupled plasma atomic emission spectroscopy (Agilent 720ES, USA). All of the analyses were carried out in triplicate.

### Porosity development

The biochar surface area was determined using a surface area analyzer (ASAP2020, Micromeritics, USA) based on the nitrogen adsorption principle, and was calculated by employing the Brunauer–Emmet–Teller (BET) equation. SEM (S-3400N, Hitachi, Japan) was used to examine the phase development and microstructure on the surface of the sludge biochar particles. The SEM was operated at 15 kV acceleration voltages, and the samples were gilded with Au and stored in a vacuum desiccator until further analysis.

### Functional groups, microcrystal structure, and chemical structure

The contents of elemental carbon, hydrogen, nitrogen, and sulfur in the sludge biochar were determined using the element analyzer (Vario EL III, Germany), and the oxygen content was calculated by difference. The X-ray fluorescence (XRF) spectroscopy (S4 Explorer, Bruker-Axs Co., Germany) was used for the quantification of the inorganic constituents of the sludge biochar.

The FT-IR technique was used for qualitative estimation of the functional groups on the surface of the sludge biochar. FT-IR spectroscopy (Nicolet 5700, USA) was performed using the potassium bromide (KBr) pellet method. The spectra for all of the samples were obtained by subtracting the value obtained from the blank sample.

The XRD patterns were recorded using an X-ray diffractometer (Bruker D8 Advance Series 2, Bruker Co., Germany) to monitor the mineral composition and degree of crystallinity on the surface of the sludge and its biochar. A quartz standard slide was run to check for instrument wander and obtain an accurate location of 2θ peaks.

The Raman technique was used to further analyze the graphite-like microstructure evolution of the carbon materials. Raman spectroscopy analysis was conducted using a visible Raman system (LabRam-1B, JY Co., France) with a 15 mV, 632.8 nm He-Ne laser.

The XPS experiments were carried out on an RBD upgraded PHI-5000CESCA system (Perkin Elmer) with Mg Kα radiation (hν = 1253.6 eV) or Al Kα radiation (hν = 1486.6 eV) to describe the evolution of the nitrogen forms. Any binding energy correction for the biochar samples was made by assigning a binding energy of 284.6 eV to the principal C1s component, and the fitting procedure was based on the FWHM of 1.8 eV.

## Supplementary Material

Supplementary Information supplementary information

## Figures and Tables

**Table 1:** The physicochemical characteristics and elemental compositions of the sewage sludge (dry basis) and sludge biochar derived (dry basis) at various pyrolysis temperatures

Items	Temperature (°C)							
	Sludge	300	400	500	600	700	800	900
Yield (%)	100	64.28	56.55	55.26	53.41	46.66	42.85	42.22
Heating value (kJ kg^−1^)	13674.3	11054.3	9988.7	8843.5	6635.6	6630.3	6587.3	6515.2
Ash (wt%)	32.80	37.42	49.17	57.42	63.24	66.66	68.32	71.18
Volatile Matter (wt%)	61.91	46.83	36.91	30.05	21.43	14.65	11.07	7.57
Fixed carbon (%)	5.29	15.75	13.92	14.53	15.32	18.68	20.61	21.25
Carbon (wt%)	33.18	37.15	35.02	30.08	26.48	27.23	26.77	25.23
FC/C (%)	15.9	42.4	39.7	48.3	57.8	68.6	77.0	84.2
Hydrogen (wt%)	5.17	4.35	2.99	1.68	1.12	0.89	0.72	0.64
Nitrogen (wt%)	5.35	6.17	4.96	4.32	3.54	3.08	2.50	1.24
Sulfur (wt%)	1.01	1.54	0.71	0.58	0.33	0.51	0.45	0.55
Oxygen (wt%)	22.49	13.37	7.15	5.91	5.29	1.63	1.24	1.16
H/C atomic ratio	1.87	1.41	1.02	0.67	0.51	0.39	0.32	0.30
O/C atomic ratio	0.51	0.27	0.15	0.15	0.15	0.04	0.03	0.03
pH	6.2	6.2	7.5	8.1	10.8	11.9	11.7	9.4
EC (ds m^−1^)	4.7	3.3	0.4	0.5	0.3	1.3	0.7	0.4
Surface area (m^2^ g^−1^)	2.88	4.88	7.56	10.79	12.22	18.28	19.11	34.21

**Table 2:** The Si, Al, and Fe contents in the ash of raw sludge and its sludge biochar samples

Inorganic matters (%)	Temperature (°C)							
	Sludge	300	400	500	600	700	800	900
Si	19.1	24.1	22.6	26.8	18.0	23.4	20.8	22.7
Al	6.9	8.7	8.2	9.3	5.9	8.0	6.9	7.3
Ca	5.7	8.1	7.7	8.0	6.5	6.9	6.8	6.4
Fe	5.1	6.1	6.1	6.2	5.4	5.4	5.5	5.4

**Table 3:** Total contents of nutrients and trace metals in raw sludge and sludge biochar samples by acid-extraction method

Items (mg g^−1^)	Sludge	Temperature (°C)													
		300	Raised (%)	400	Raised (%)	500	Raised (%)	600	Raised (%)	700	Raised (%)	800	Raised (%)	900	Raised (%)
P	7.74	10.43	34.7	11.56	49.3	16.63	114.8	18.23	135.5	20.01	158.5	18.98	145.2	19.48	151.6
K	2.05	2.25	9.7	2.48	21.0	2.75	34.1	2.83	38.0	2.91	41.9	3.43	67.3	3.35	63.4
Na	3.49	3.50	0.3	4.25	21.8	4.38	25.5	7.40	112.0	8.11	132.4	6.70	92.0	6.41	83.7
Ca	5.00	5.33	6.6	5.59	11.8	6.00	20.0	6.45	29.0	7.80	56.0	8.55	71.0	9.14	82.8
Mg	1.25	1.35	8.0	1.42	13.6	1.68	34.4	2.24	79.2	2.56	104.8	2.85	128.0	3.19	155.2
Al	1.60	1.77	10.6	3.40	112.5	3.89	143.1	4.38	173.7	5.50	243.7	5.89	268.1	6.06	278.7
Fe	1.10	1.26	14.5	2.20	100.0	2.50	127.3	2.55	131.8	2.98	170.9	2.80	154.5	3.36	205.4
Zn	0.81	0.91	12.3	1.11	37.0	1.58	95.1	1.72	112.3	1.78	119.8	1.70	109.9	1.73	113.6
Mn	0.27	0.28	3.7	0.30	11.1	0.42	55.6	0.48	77.8	0.57	111.1	0.20	−25.9	0.11	−59.2
Cu	0.11	0.09	−18.2	0.09	−18.2	0.18	63.6	0.14	27.3	0.16	45.5	0.09	−18.2	0.09	−18.2

**Table 4:** Contents of water-extractable compounds in raw sludge and sludge biochar samples

Items (mg g^−1^)	Temperature (°C)							
	Sludge	300	400	500	600	700	800	900
DOC	31.24	24.23	2.66	0.32	0.16	0.15	0.08	0.03
DN	8.90	6.19	0.57	0.07	0.06	0.06	0.03	0.02
NH_4_^+^-N	5.57	4.39	2.15	BD	BD	BD	BD	BD
P	1.39	1.00	0.21	BD	BD	BD	BD	BD
K	1.36	0.73	0.15	0.17	0.15	0.11	0.16	0.26
Na	1.62	0.25	0.20	0.18	0.18	0.18	0.18	0.17
Ca	1.90	0.51	0.49	0.47	0.87	1.28	1.41	0.50
Mg	0.60	0.53	0.21	0.18	BD	BD	BD	BD
Al	BD	BD	BD	BD	BD	BD	BD	BD
Fe	BD	BD	BD	BD	BD	BD	BD	BD
Zn	BD	BD	BD	BD	BD	BD	BD	BD
Mn	BD	BD	BD	BD	BD	BD	BD	BD
Cu	BD	BD	BD	BD	BD	BD	BD	BD

BD: below the detected limit.

**Table 5:** XPS Integrated areas (%) of N regions corresponding to their binding energies for raw sludge and sludge biochar samples

Binding type	Binding energy (eV)	Temperature (°C)							
		Sludge	300	400	500	600	700	800	900
N-6	398.7	11.1	34.8	59.0	57.1	53.6	54.9	44.4	40.4
N-5	400.4	61.1	65.2	39.8	36.8	38.8	35.7	43.3	38.3
N-Q	401.1	27.8	0	1.2	6.1	7.6	9.4	12.3	21.3
