# Supplementary material for: Multiscale visualization of the structural and characteristic changes of sewage sludge biochar oriented towards potential agronomic and environmental implication
Source: Sci Rep. 2015 Mar 24;5:9406. doi: 10.1038/srep09406 (PMC4371148; doi:10.1038/srep09406)

Multiscale visualization of the structural and characteristic changes of sewage sludge biochar oriented towards potential agronomic and environmental implication

Jining Zhang, Fan Lü, Hua Zhang, Liming Shao, Dezhen Chen, Pinjing He

**information: Table.S1 Contents of heavy metals in raw sludge and sludge biochar samples using XRF technique.**

| Items  (%) | Temperature (^o^C) | | | | | | | |
| --- | --- | --- | --- | --- | --- | --- | --- | --- |
|  | Sludge | 300 | 400 | 500 | 600 | 700 | 800 | 900 |
| Al | 2.25 | 3.26 | 4.03 | 5.35 | 3.76 | 5.32 | 4.72 | 5.19 |
| Fe | 1.66 | 2.29 | 2.99 | 3.57 | 3.41 | 3.62 | 3.77 | 3.82 |
| Cu | 0.037 | 0.032 | 0.037 | 0.042 | 0.041 | 0.044 | 0.045 | 0.047 |
| Mn | BD | 0.059 | 0.082 | 0.101 | 0.099 | 0.096 | 0.102 | 0.104 |
| Zn | 0.022 | 0.057 | 0.088 | 0.143 | 0.137 | 0.131 | 0.158 | 0.081 |

BD: below the detected limit.

**information: Fig.S2** XRD spectra of the sewage sludge and sludge biochar samples obtained at various temperature.


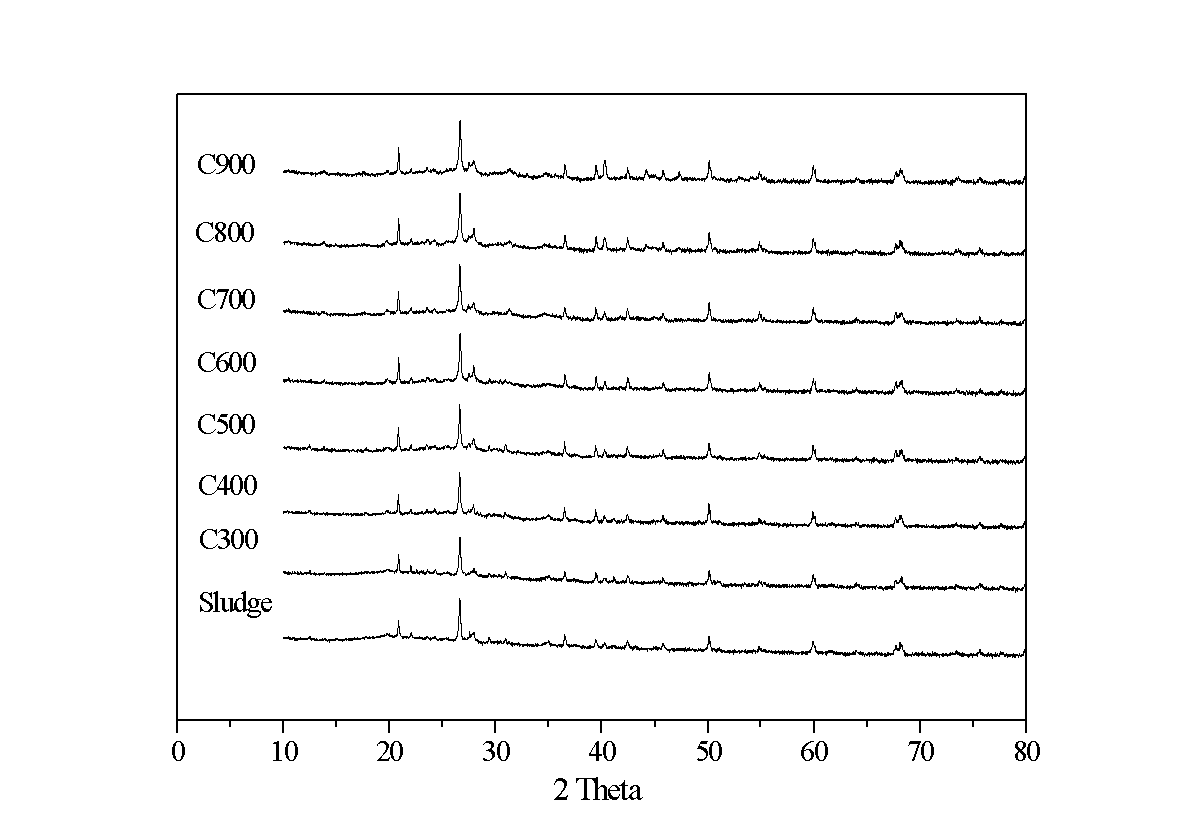


**Supplemental information: Fig.S3** SEM morphology of the sewage sludge and sludge biochar samples at various temperature. (a) primary sludge; (b) C300; (c) C400; (d)C500; (e)C600; (f)C700; (g)C800; (h)C900


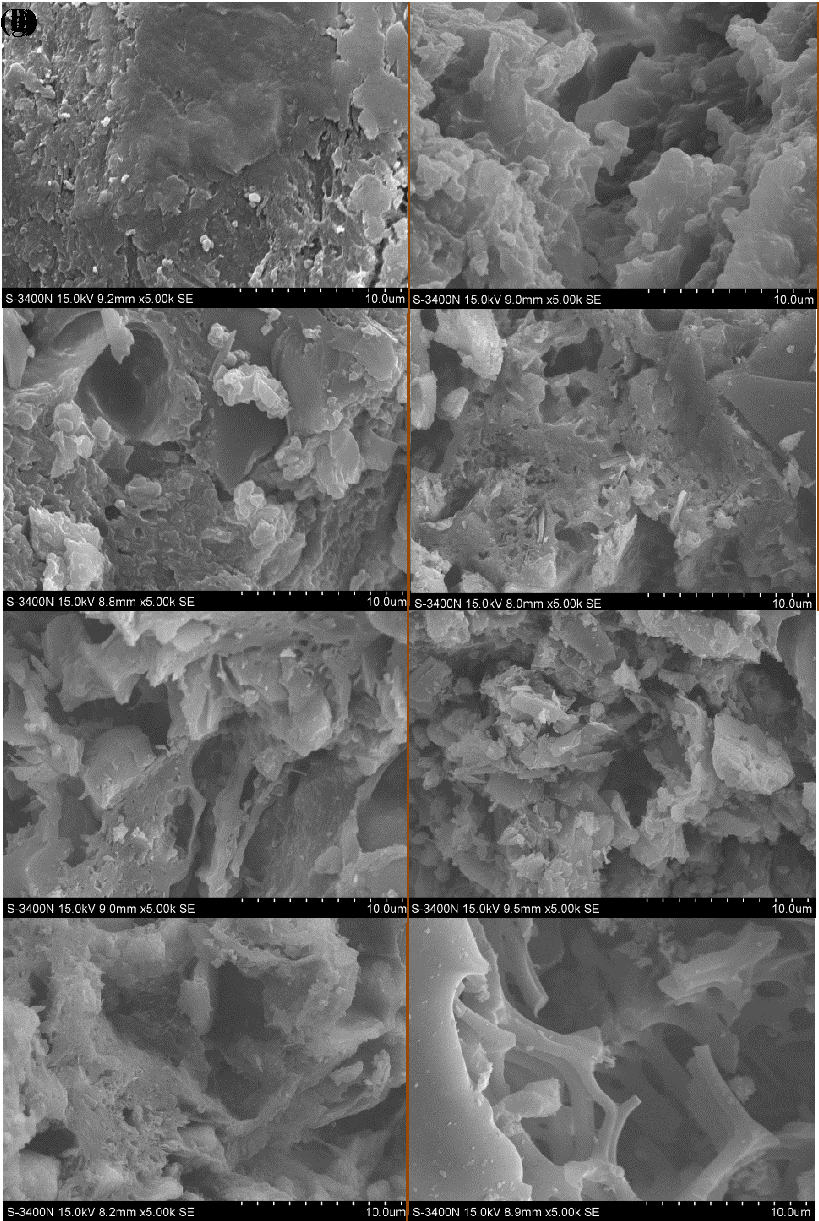


**Supplemental information: Fig.S4** FT-IR spectra of the sewage sludge and sludge biochar samples obtained at various temperature.
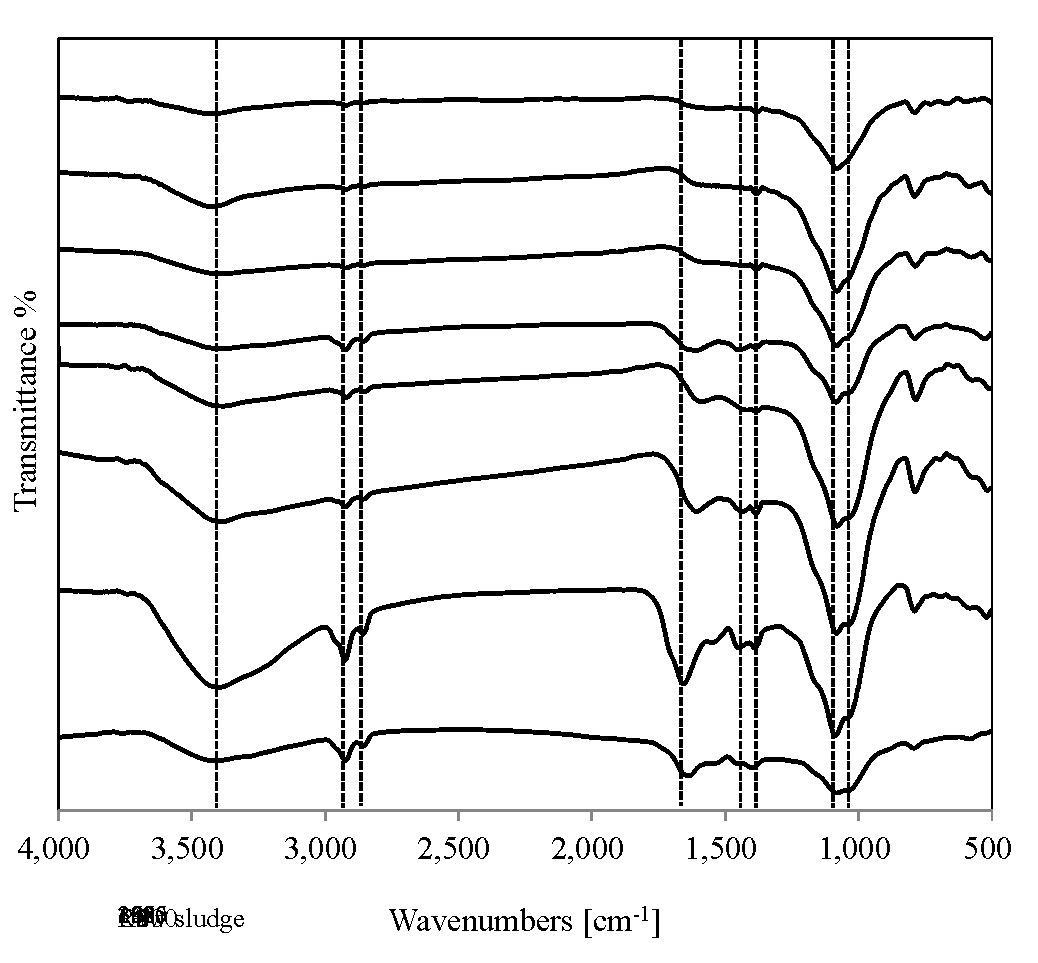


**Supplemental information: Fig.S5** Raman spectra of the sludge biochar samples obtained at various temperatures. \
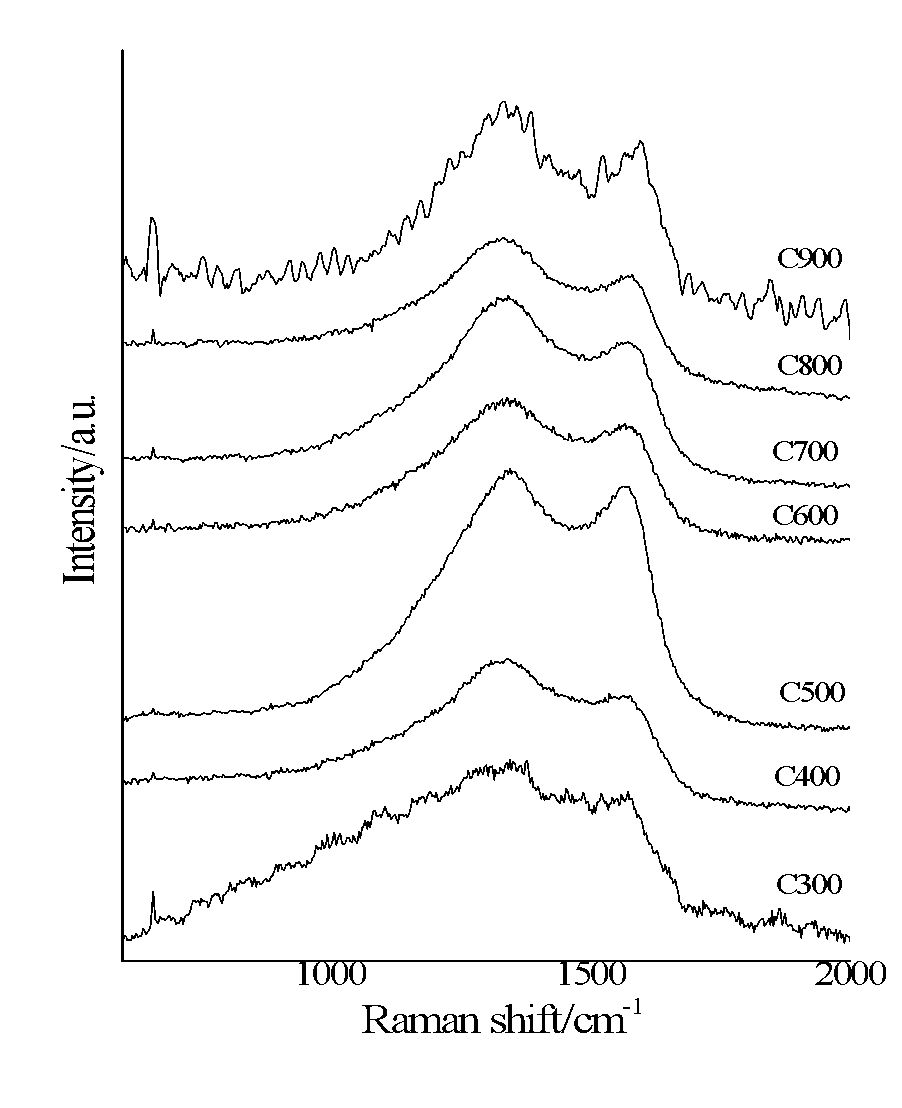

Supplement: Supplementary Information [file srep09406-s1.docx]
